# Gas-Mediated Intestinal Microbiome Regulation Prompts the Methanol Extract of *Schizonepetae Spica* to Relieve Colitis

**DOI:** 10.3390/nu15030519

**Published:** 2023-01-19

**Authors:** Xuewei Ye, Yingxin Cen, Kefei Wu, Langyu Xu, Jiahui Ni, Wenxin Zheng, Wei Liu

**Affiliations:** 1 Department of Basic Medical Sciences, Shulan International Medical College, Key Laboratory of Pollution Exposure and Health Intervention of Zhejiang Province, Zhejiang Shuren University, Hangzhou 310015, China; 2 Institute of Plant Protection and Microbiology, Zhejiang Academy of Agricultural Sciences, Hangzhou 310021, China

**Keywords:** *Schizonepetae Spica*, ulcerative colitis, intestinal flora, *Desulfovibrio*, H_2_S

## Abstract

Intestinal dysbiosis plays an important role in the pathogenesis of colitis (UC). *Schizonepetae Herba* can achieve anti-inflammatory effects as a medicine and food homologous vegetable. Luteolin, eriodictyol, fisetin, and kaempferol are the main anti-inflammatory active compounds obtained through mass spectrometry from the methanol extract of *Schizonepetae Spica* (JJSM). JJSM intervention resulted in attenuated weight loss, high disease-activity-index score, colon length shortening and colonic pathological damage in DSS-induced colitis mice. Interestingly, hydrogen sulfide (H_2_S) was inhibited remarkably, which is helpful to elucidate the relationship between active substance and intestinal flora. Furthermore, JJSM administration improved intestinal flora with down-regulating the abundance of harmful bacteria such as *Clostridiales* and *Desulfovibrio* and up-regulating the abundance of beneficial bacteria such as *Muribaculaceae* and *Ligolactobacillus* and enhanced the production of SCFAs. It is worth noticing that *Desulfovibrio* is related to the production of intestinal gas H_2_S. The elevated levels of *Desulfovibrio* and H_2_S will hasten the onset of colitis, which is a crucial risk factor for colitis. The results displayed that JJSM could considerably ameliorate colitis by rebuilding H_2_S-related intestinal flora, which provides a new therapeutic strategy for *Schizonepetae Spica* to be utilized as a functional food and considered as an emerging candidate for intestinal inflammation.

## 1. Introduction

As a non-specific inflammatory bowel condition, ulcerative colitis (UC) has plagued human health for many years, and the onset age has become younger. It is characterized by dysregulation of immune responses associated with the intestinal flora [1], leading to the perpetuation of intestinal inflammatory processes. Importantly, as the disease progresses, the risk of colorectal cancer also increases [2]. Long-term use of western medicines such as aminosalicylic acid preparations, immunosuppressants and hormones have been associated with drug resistance and adverse reactions [3,4]. Environmental variables, intestinal microbiota imbalance, aberrant immunological responses and genetic susceptibility have all been linked to UC [5]. At present, it is generally believed that gut dysbiosis may be triggered by environmental factors such as diet. The relationship between food and intestinal microbiota has gradually become a research hotspot. Medical and edible food can affect the mechanism of action in ulcerative colitis by modulating gut microbes. For example, pomegranate peel and its polyphenol-rich extract inhibit various inflammatory diseases, including ulcerative colitis. Its active ingredient urolithin A upregulates the epithelial tight junction protein by stimulating the aryl hydrocarbon receptor (AhR) nuclear factor-erythroid 2 related factor 2 (Nrf2)-dependent pathway, improving intestinal barrier function and inhibiting inflammation [2,6]. Therefore, dietary therapy may become a new strategy for treating ulcerative colitis.

Natural plants, especially medicine food homology and functional foods, are increasingly used, for instance, *Schizonepetae Spica*, the dried flower spike of *Schizonepetae Herba* [7,8]. Traditional Chinese medicine believes that the incidence of UC is related to dampness, and it has been found that the windproof effect of *Schizonepetae Herba* can significantly improve the symptoms of abdominal pain, diarrhea, pus and bloody stool in patients with colitis [9,10]. According to the Chinese Materia Medica, *Schizonepetae Herba* acts on the hemostatic process and is windproof. It is mainly used for fever, vomiting blood, hematochezia and other symptoms. An increasing body of evidence suggests that JJSM extracts exert anti-inflammatory effects by preventing the activation of the Toll-like receptor 4 (TLR4) and proinflammatory cytokines in LPS-stimulated macrophages [11].

In this study, the influence of the methanol extract of *Schizonepetae Spica* (JJSM) on weight, disease activity index (DAI) score, colon histomorphology, gut microbial composition, gas and short-chain fatty acid (SCFA) production were evaluated in dextran sulfate sodium (DSS)-induced colitis mice. Of note, JJSM intake could reduce colon damage and correct the intestinal microecological imbalance and the metabolites with H_2_S-producing bacteria (*Desulfovibrio*) to investigate the mechanism underlying symptom relief in DSS-induced colitis mice. The present findings may provide potential new strategies for the treatment of colitis.

## 2. Materials and Methods

### 2.1. Preparation of Plant Extracts

*Schizonepetae Spica* was purchased from Bozhou Siyuantang Traditional Chinese Medicine Sales Co., Ltd. (Anhui, China). The dry powder of *Schizonepetae Spica* was extracted three times with 100% methanol at room temperature and then filtered to obtain the primary extract. These filtrates were concentrated under reduced pressure and combined to obtain the methanol extract of *Schizonepetae Spica* (JJSM). JJSM was concentrated to dryness in a vacuum and stored at 4 °C. The organic solvents of JJSM were evaporated with a vacuum concentrator, and the remaining water was removed by freeze drying. After drying, the JJSM sample was stored at 4 °C.

### 2.2. Chemical Composition Analysis of JJSM

Based on ultra-high performance liquid chromatography q exactive mass spectrometry (UHPLC-QE-MS), JJSM was detected in a non-targeted manner using an ACQUITY UPLC^®^ HSS T3 1.8 m (2.1 mm × 150 mm) column. The mobile phase consisted of 0.1 percent formic acid water (C)–0.1 percent formic acid acetonitrile (D) in a positive ion mode and 5 mM ammonium formate water (A)–acetonitrile (B) in a negative ion mode. Configuration of the gradient elution program was as follows: 0~1 min, 2% B/D; 1~9 min, 2~50% B/D; 9~12 min, 50~98% B/D; 12~13.5 min, 98% B/D; 13.5~14 min, 98~2% B/D; 14~20 min, 2% D-positive mode (14~17 min, 2% B- negative mode) [12].

### 2.3. Experimental Design

Six-week-old male specific pathogen-free (SPF) BABL/c mice (40) were purchased from the Animal Experiment Center of Hangzhou Medical University and randomly assigned to five groups (*n* = 8 in each group): blank control group (Control), model group (Model), positive control group (Salazosulfapyridine, SASP), JJSM low concentration group (JJSM-L) and JJSM high concentration group (JJSM-H). The mice were kept in sterile environments with a regulated temperature and humidity cycled every 12 h [13] under temperature-controlled and sterile conditions. Mice in the control group were given free access to sterile water, whereas mice in the other groups were given free access to 3% DSS solution (replaced every two days) for 8 days to develop acute experimental colitis. Groups were respectively given intragastric administration with normal saline, 200 mg/kg SASP [14,15], 500 mg/kg JJSM and 1000 mg/kg JJSM [16] for 8 days. The body weight, softness of feces and anal bleeding of the mice were recorded, and the drug intervention was observed after daily intragastric administration.

### 2.4. Evaluation of Anti-UC Effects

The DAI score is a popular metric for assessing colonic injury models in experimental animals. DAI-related traits, including body weight, stool consistency, and blood in the stool, were observed and recorded daily, and DAI was scored according to the following parameters [17]: Weight loss (0: None; 1: 1–5%; 2: 5–10%; 3: 10–20%; 4: >20%), stool consistency (0: Normal; 2: Loose droppings; 4: Diarrhea) and blood in the stool (0: Normal; 2: Occult blood positive; 4: Blood bleeding), taking the average of the three. The mice were slaughtered on the eighth day of the experiment to examine changes in colon edema, adhesion, ulcer, necrosis, etc., and the length of the colon was measured with a ruler and photographed, and the gross morphology of the colon was analyzed. The efficacy of JJSM on UC was preliminarily determined based on the following two indicators [18].

### 2.5. Colon Lesion Assessment

The harvested distal colons of each experimental group were sliced, and the pathological evaluation of the colon lesions of the mice in each group was performed. A 0.5 cm piece of colon tissue was embedded in paraffin after being treated in 4% paraformaldehyde for 48 h. Hematoxylin and Eosin (H&E) was used to stain 5 µm–thick sections [19]. Colon tissue damage was observed under light microscopy at different magnifications, and histopathological scoring was performed based on seven aspects [17]: Degree of inflammation (score ranged from 0~4 for Normal, Mucosal, Submucosal, Muscular and Serous involvement), Degree of damage to the crypt (score ranged from 0~4 for Normal, basal 1/3 of the crypt, basal 2/3 of the crypt, entire crypt involvement and damage to the crypt and ulceration), Crypt abscesses (score ranged from 0~2 for Normal, Unifocal and Multifocal involvement), Infiltration of inflammatory cells, Degree of submucosal edema, Goblet cell reduction, Degree of epithelial hyperplasia (score ranged from 0~3 for Normal, Unifocal, Multifocal and Diffuse involvement).

### 2.6. Analysis of the Intestinal Microbiota

#### 2.6.1. SCFA Production

After the experiment, the fecal broth was centrifuged at 10,000 r/min for 10 min at 4 °C; the supernatant was extracted, mixed with the internal standard crotonic acid at a ratio of 5:1, and refrigerated and acidified for more than 24 h. Finally, it was used for gas chromatography to measure the amounts of acetic acid, propionic acid, butyric acid, isobutyric acid, valeric acid and isovaleric acid in fecal bacteria fermentation acidification solution [19].

#### 2.6.2. Gas Production

Batch culture fermentations were performed according to reported methods. The medium contained the following ingredients in grams per liter form (g/L): soluble starch, 8; tryptone, 6; yeast extract, 4.5; NaCl, 4.5; KCL, 2.5; L-cysteine hydrochloride, 0.8; MgCl_2_·6H_2_O, 0.45; bile salt, 0.4; KH_2_PO_4_, 0.4; CaCl_2_·6H_2_O, 0.2; Hemin, 0.05; 1 mL of Tween-80 and 2 mL of a solution of trace elements (g/L: MgSO_4_·7H_2_O, 3.0; MnCl_2_·4H_2_O, 0.32; FeSO_4_·7H_2_O, 0.1; CoSO_4_·7H_2_O, 0.18; CaCl_2_·2H_2_O, 0.1; ZnSO_4_·7H_2_O, 0.18; CuSO_4_·5H_2_O, 0.01 and NiCl_2_·6H_2_O, 0.092). The medium pH was adjusted to 6.5 and sterilized [20].

A total of 0.24 g of fresh stool samples were homogenized with 2.4 mL of 0.1 M anaerobic phosphate-buffered saline (pH 6.5). Then, 0.2 mL of fecal slurry and 5 mL of culture medium were transferred to 10 mL bottles with different groups (Control, Model, JJSM-L, JJSM-H group) and vortexed for 10 s. The bottles were incubated for 24 h at 37 °C. The gas composition was measured using the fermentation gas analyzer to simultaneously detect carbon dioxide (CO_2_), hydrogen (H_2_), methane (CH_4_), hydrogen sulfide (H_2_S) and ammonia (NH_3_). A needle-tipped gas-impermeable syringe was inserted into the rubber cap of each vessel to measure the concentration of each gas produced.

#### 2.6.3. Microbial Diversity

16S rDNA sequencing was performed on each stool sample. DNA was extracted from the fermentation broth and detected by electrophoresis. Then, Polymerase Chain Reaction (PCR) amplification was performed on the V3-V4 region of the sample 16S rRNA using primers. Amplicons were quantified and combined, library preparation and quality control (QC) were performed, and sequencing was done using an Illumina MiSeq machine. After the on-machine sequencing was completed, we obtained the original off-machine data RawData. The double-ended data were joined using overlap after quality assurance and chimera filtering to create high-quality data. The Divisive Amplicon Denoising Algorithm (DADA2) was used to obtain representative sequences with single-base precision rather than grouping based on sequence similarity through processes like “dereplication” (equal to clustering with 100% similarity). The precision resolution and data accuracy were both considerably enhanced. Further diversity analysis, annotation of species categorization, difference analysis, and correlation analysis were carried out after obtaining the final ASV feature table and feature sequence. Following trimming, paired-end reads were first put together using FLASH software, and chimera-containing reads were found and removed using UCHIME software. Clean reads were clustered into OTUs selected by the Uparse software27 (version 7.0.1001) with a similarity threshold of 97%. Furthermore, alpha diversity was assessed by calculating Goods_coverage, Chao1 and Shannon diversity indices. Then, the beta diversity was assessed by NMDS and cluster tree analysis to observe similarities and differences between individuals or groups. As for the LDA Effect Size (LEfSe) analysis, linear discriminant analysis (LDA) scores were used to describe the differences among the five groups of mice.

### 2.7. Statistical Analysis

For statistical analysis of the acquired data, SPSS 26.0 software was employed. The data were reported as mean ± standard (S.D) deviation of at least three separate experiments. One-Way ANOVA was used to examine the variables. A *p*-value < 0.05 was statistically significant.

## 3. Results and Discussion

### 3.1. Identification of Chemical Constituents in JJSM

UHPLC-QE-MS was used to identify the 354 compounds in JJSM. Based on the screening criteria oral bioavailability (OB) > 30% and drug-like properties (DL) > 0.18 from the traditional Chinese medicine systems pharmacology (TCMSP) database, nine bioactive compounds, including seven flavonoids, one isoflavone, one benzene and a substituted derivative were obtained. Table 1 shows the retention time (RT), *m*/*z*, discriminant component, OB value, DL value and category. Four compounds (luteolin [21,22], eriodictyol [23], fisetin [24] and kaempferol [25]) contained in JJSM may alleviate inflammatory bowel disease (IBD) and even potentially prevent against Colon and Rectal Cancer (CRC) based on previous reports. It has been shown that chronic IBD may develop into colorectal cancer. There are three main types of tumor microenvironments: hypoxia, chronic inflammation and immunosuppression. Luteolin, one of the important compounds in JJSM, has been shown to have anticancer properties. Current evidence suggests that luteolin downregulates pleiotrophin (PTN) through miR-384 expression, a potent tumor suppressor molecule that exerts anticancer effects on CRC cells [26]. Prophylactic use of luteolin in a model of intestinal mucositis could attenuate intestinal wall damage and alleviate diarrhea and weight loss following peroxisome proliferator-activated receptor γ (PPAR-γ), and effectively alleviate typical symptoms of intestinal inflammation [27]. Moreover, it has been documented that eriodictyol significantly inhibits the inflammatory response by modulating mediators and inhibiting the TLR4/NF-κB signaling pathway, significantly reducing the number of different inflammatory cells. Although there is no direct evidence for its effects on the gut, it can protect nerves, kidneys and lungs by reducing the production of inflammatory cytokines and increasing the activity of antioxidant enzymes [23]. It is speculated that NF-κB signaling and the release of proinflammatory mediators may be reduced to some extent, thereby alleviating inflammatory bowel disease. Fisetin acts as a dietary flavonoid in PI3K/AKT/mTOR signaling. It has been shown that Fisetin could inhibit PI3K/AKT and mTOR pathways, induce autophagy in human cancer cells [28] and inhibit distal colon tumor development, further preventing the occurrence of intestinal tumors in mice [29]. Kaempferol, another component of JJSM, is also known to be one of the most active and important natural anti-inflammatory flavonoids [30]. Kaempferol can reportedly downregulate the expression of cyclin-dependent kinases 2(CDK2), CDK4 and other oncogenes and induce the phosphorylation of the ERK-1/2 signaling pathway [31]. In addition, ROS and p53 signaling pathways mediate p38 phosphorylation and caspase activation of kaempferol and induce apoptosis of CRC cells, indicating the anticancer potential of kaempferol [32]. JJSM has potential anti-tumor characteristics that prevent or delay chronic UC.

### 3.2. JJSM Reduces the Disease Activity Index in Mice

**Figure 1:**
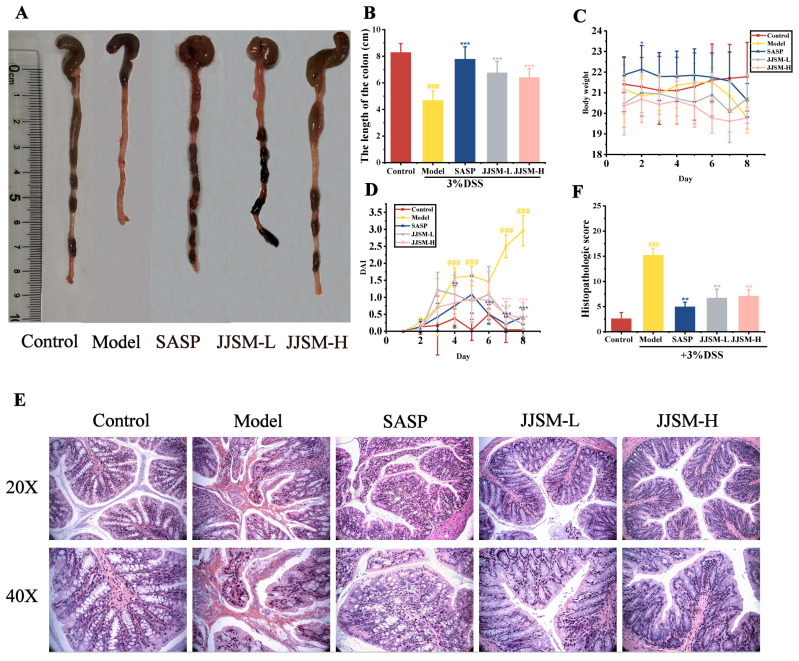
JJSM ameliorated the clinical symptoms of DSS-induced colitis in mice. (**A**) Macroscopic view of colon length; (**B**) Colon length; (**C**) Changes in body weight; (**D**) DAI scores. Effect of JJSM on histopathological changes. (**E**) Hematoxylin and Eosin (H & E)-stained representative colonic tissues; (**F**) Histological score. Data are presented as the mean ± S.D. ^###^ *p* < 0.001 vs. Control group; * *p* < 0.05, ** *p* < 0.01 vs. Model group, *** *p* < 0.001 vs. Model group.

Colon length is one of the main parameters for evaluating the severity of colitis. The colon lengths were 8.25 ± 0.71 cm, 4.66 ± 0.74 cm, 7.75 ± 0.96 cm, 6.72 ± 0.90 cm and 6.38 ± 0.69 cm in the Control, Model, SASP, JJSM-L and JJSM-H groups. The measured values of other groups were significantly longer than the Model group. As indicated in Figure 1A, there was a significant difference in colon length between the control and Model groups (*p* < 0.001). The colon lengths of the Model group, SASP group, JJSM-L group and JJSM-H group were reduced by about 43.52%, 6.06%, 18.55% and 22.67%, respectively, compared to the Control group (Figure 1A,B). Additionally, the colon of the model group of mice exhibited various ulcer-related lesions and thickening of the intestinal wall. However, the colon and intestinal wall of the JJSM intervention groups were relatively intact, without obvious ulcers, and the length of the colon was prolonged to varying degrees.

Eight days after the injection, the DSS-induced colitis mice displayed overt clinical signs, such as weight loss, diminished vitality, fluffy hair, diarrhea, and fecal occult blood or bleeding in stools, which accounted for the significantly higher DAI score than normal mice. However, in the JJSM drug intervention groups mice, no significant blood was found in the feces with relief of clinical symptoms, resulting in a lower DAI (Figure 1C,D). These preliminary findings suggest that JJSM could significantly reduce the colitis caused by DSS.

### 3.3. JJSM Improved the Pathological Conditions in Mice with Colitis

Histopathological damage in colon tissue was assessed using H&E staining. In the control group, the layers of colon tissue were clearly visible; the mucosal epithelium was intact and continuous, the glands were regularly arranged, no inflammatory cell infiltration and no ulcers were found (Figure 1E). The superficial layer of the colonic mucosa in the model group had ulcers, bleeding was visible, and the structure of the mucosal layer was disrupted, with significant infiltration of inflammatory cells. In addition, there was submucosa edema, which was of inflammatory origin, with no visible goblet cells. The damaged area of the colon was significantly larger, and the histopathological score was significantly higher than the control group (Figure 1F). The colon mucosa structure of the SASP group and JJSM drug intervention groups improved to varying degrees, without significant ulcer formation and infiltration of inflammatory cells and reduced crypt damage. The JJSM intervention groups dramatically decreased the histology score compared to the model group, which indicated that JJSM alleviated colitis-related colonic injury.

### 3.4. JJSM Altered the Production of Intestinal Gas

It has been established that gas levels in IBD (inflammatory bowel disease) and colon cancer differ from healthy subjects [33]. Gas is produced in the stomach by microbiota and enzymes that ferment indigestible carbohydrates [34]. Measuring intestinal gas can reveal the impact of intestinal microbiota metabolites and metabolite-related functions, which is critical in the pathogenesis of gastrointestinal inflammation. During fermentation, we monitored gas pressure to indicate the fermentation pace and gas composition.

**Figure 2:**
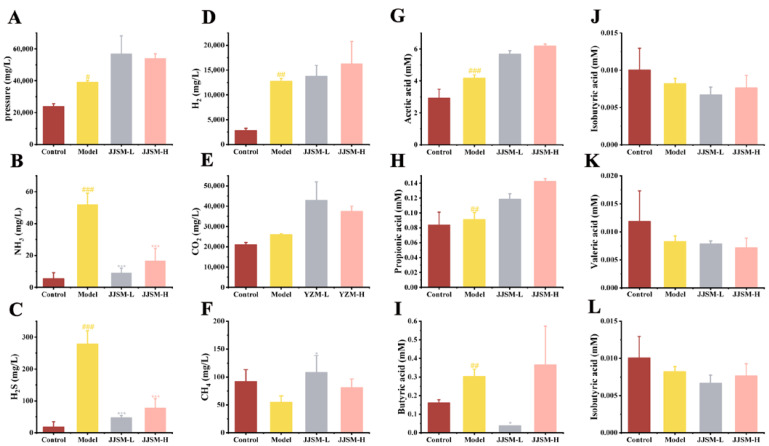
Gas production. (**A**) The pressure that remains after fermentation. *Schizonepetae Spica* results in increased pressure after incubation; (**B**) NH_3_; (**C**) H_2_S; (**D**) H_2_; (**E**) CO_2_; (**F**) CH_4_. The x-axis displays the sample groups, the y-axis displays gas amount, the gas abundance value is the mean of three biological replicates, and the error bars reflect the 95% confidence intervals. Short-chain fatty acid (SCFA) production. (**G**) Acetic acid; (**H**) Propionic acid; (**I**) Butyric acid; (**J**) Isobutyric acid; (**K**) Valeric acid; (**L**) Isovaleric acid. Data are presented as the mean ± S.D. ^#^ *p* < 0.05 vs. control, ^##^ *p* < 0.01 vs. control, ^###^ *p* < 0.001 vs. control, * *p* < 0.05 vs. model, *** *p* < 0.001 vs. model. M: molecular weight of gas.

As shown in Figure 2, the predominant gas in the Control group was CO_2_, followed by H_2_, H_2_S and NH_3_. The gas production pressure (Figure 2) in JJSM intervention groups was considerably greater than in normal mice and model mice, indicating that JJSM had a stimulating effect on bacterial gas production, and the effect was more obvious in the low concentration group. High levels of H_2_S prevent the body from properly oxidizing butyrate and other short-chain fatty acids, which can result in nutritional deficiencies [33]. Moreover, high concentration of H_2_S is potentially toxic to tissues [35]. In our research, the content of H_2_S in mice with colitis was significantly increased (*p* < 0.001), also suggesting that it was toxic to mice and exacerbated colitis. NH_3_ and H_2_S gas production in the Model group was high, while the JJSM intervention groups showed significant inhibitory effects on these two gases (*p* < 0.001), and the two gas production trends were comparable (Figure 2B,C).

Significant H_2_ gas production was observed in mice after modeling (*p* < 0.01) (Figure 2D), whereas the improvement effect of JJSM drug intervention groups was not obvious. There was no significant difference in CO_2_ production between colitis mice and normal mice (Figure 2E). In addition, the CH_4_ gas production in the JJSM-L group was substantially greater than in the Model group (*p* < 0.05), while CH_4_ gas production in the JJSM-H group was not inferior to the Control group. However, due to the low proportion of CH_4_ in the total gas production, the overall effect made little difference (Figure 2F).

### 3.5. JJSM Changed the Production of SCFAs

It has been demonstrated that acetic acid helps maintain the integrity of the intestinal barrier and provides energy to intestinal epithelial cells [36]. Acetate [37] and butyrate [38] have been shown to enhance intestinal epithelial barrier function and protect against pathogen invasion. SCFA assays were also performed in our research (Figure 2).

The model group’s intestinal acetic acid, propionic acid and butyric acid levels were significantly higher than the Control group (*p* < 0.01). The intestinal microbial metabolism of mice in the JJSM intervention groups mainly produced a large amount of acetate. Compared with the Control group (2.93 mmol/L), the acetate concentration in the JJSM intervention groups changed dose-dependently, increasing to 5.67 mmol/L in the JJSM-L group and 6.18 mmol/L in the JJSM-H group (Figure 2G). A consistent trend was observed for propionic and acetic acid (Figure 2H).

A certain concentration of butyric acid can relieve inflammation and improve the function of the epithelial barrier of colon cells. However, the clearance of pathogens can be reduced when the butyrate concentration is too high [39]. Excess butyric acid damaged the mice’s intestinal barrier and aggravated intestinal inflammation. A significant difference in butyric acid concentration was found between the Control group (0.16 mM) and the Model group (0.30 mmol/L) (*p* < 0.05). The results showed that the JJSM low-concentration group and high-concentration group exhibited opposite effects, and the JJSM-L group inhibited the production of butyric acid to a certain extent (*p* < 0.05), while the concentration of butyrate (0.37 mmol/L) in JJSM-H increased (Figure 2I).

The contents of acetic acid, propionic acid and butyric acid in colitis mice were significantly greater than in normal mice. Acetate [37] and butyrate [38] have been shown to enhance intestinal epithelial barrier function and protect against pathogen invasion. The butyric acid and isovaleric acid of the JJSM-L group were significantly different from the Model group (*p* < 0.05) (Figure 2L). The isobutyric acid and valeric acid levels in the colitis mice and normal mice were comparable (Figure 2J,K).

Overall, the above results suggested that JJSM still assists in the production of SCFAs in UC mice, which can enhance colonic epithelial cell shape, function, and intestinal function, consequently enhancing the acidity of the intestinal environment and controlling inflammation.

### 3.6. JJSM Regulated the Intestinal Microbiome

There is growing evidence that bacterial abundance and diversity in the gut microbiome can help prevent cell necrosis of the colon mucosa and reshape the intestinal barrier. The imbalance of intestinal flora is considered a key factor that can trigger the pathogenesis of colitis.

#### 3.6.1. Alpha and Beta Diversity of the Intestinal Microbiota

**Figure 3:**
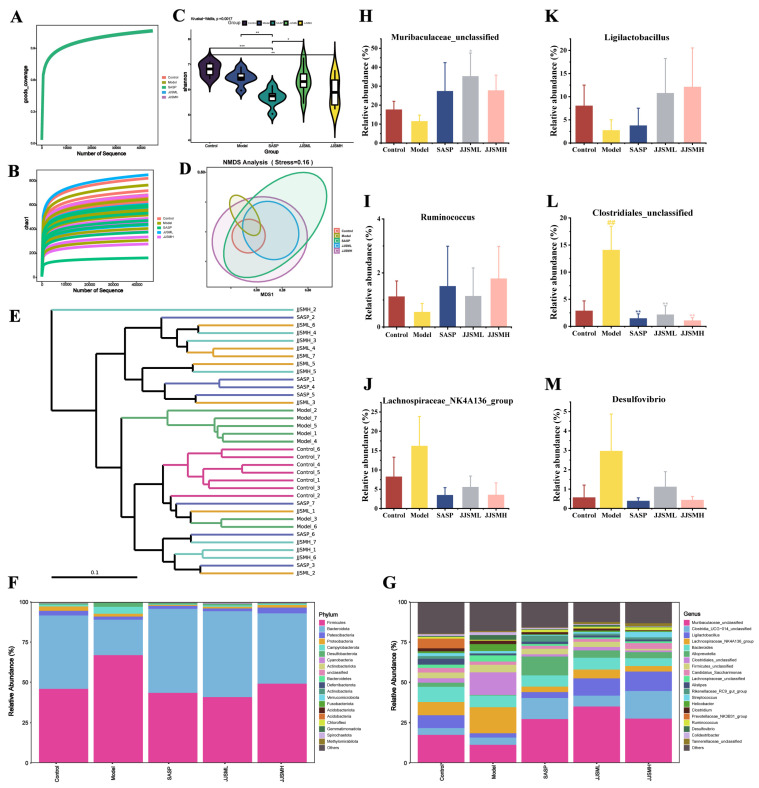
Analysis of microbial Alpha diversity. (**A**) Goods_coverage; (**B**) Chao 1 index and (**C**) Shannon index. Beta diversity: (**D**) Weighted NMDS analysis and (**E**) Weighted cluster tree analysis. Microbial community structure of each group: (**F**) Distribution of microbiome at phylum; (**G**) Distribution of microbiome at genus; (**H**) Relative abundance of *Muribaculaceae_unclassified*; (**I**) Relative abundance of *Ligilactobacillus*; (**J**) Relative abundance of *Ruminococcus*; (**K**) Relative abundance of *Clostridiales_unclassified*; (**L**) Relative abundance of *Lachnospiraceae_NK4A136_group*; (**M**) Relative abundance of *Desulfovibrio*. Data are presented as the mean ± S.D. ^##^ *p* < 0.01 vs. control, * *p* < 0.05 vs. model, ** *p* < 0.01 vs. model, *** *p* < 0.001 vs. model.

Alpha diversity refers to the diversity within a specific area or ecosystem. Goods_coverage refers to microbial coverage; the higher the value, the lower the probability that new species in the sample are not detected. As shown in Figure 3A and Table S1, a value infinitely close to 1 indicates that the sequencing results represent the real situation of the sample. Compared with the control group (Chao1 = 621, Shannon index = 6.48), the alpha diversity of the Model group (Chao1 = 511.13, Shannon index = 6.43) was reduced. Chao1 was used to determine the species richness index (Figure 3B). Lower richness indices were associated with colitis-affected mice, suggesting that DSS destroyed the microbial structure and JJSM-L improved its species diversity. The Shannon index results were displayed in a violin plot to illustrate the microbial diversity of mice from various groups, with higher values indicating higher diversity. Reduced alpha diversity was observed in the Model group, while the SASP group had a significant decrease in alpha diversity compared to the Model group, and the JJSM intervention groups showed slightly better effects than the SASP group (Figure 3C).

The Beta diversity represents the variations in the composition of microbial communities among various sample groups. This study employed non-metric multidimensional scaling (NMDS) and hierarchical cluster analysis to determine beta diversity in each group. The SASP-treated samples exhibited the largest within-group differences, while the other groups of mice had smaller within-group differences (Figure 3D). On the other hand, the flora of the Control group and Model group were clearly separated. The microbial community structure of the Model group and Control group were significantly different, with comparable bacterial composition within the groups, according to hierarchical clustering analysis of OTUs (Figure 3E). Analysis of intestinal flora showed that the composition of the intestinal flora of UC mice was altered after SASP and JJSM treatment, while the structure of intestinal flora of mice in the JJSM intervention groups was closer to that of normal mice, indicating that JJSM can regulate intestinal flora disorders and improve UC.

#### 3.6.2. Composition and Abundance of Intestinal Microbiota

To characterize the composition of the gut microbiomes of these UC mice, we analyzed fecal samples from each group. 16S rDNA amplicon sequencing was performed on each stool sample, followed by an analysis of microbial community taxonomic composition and potential function. The difference in prevalence for each group at the phylum level was shown in Figure 3F (the top 20 representative phyla). After DSS treatment, the taxa of harmful microorganisms increased, while the taxa of beneficial microorganisms decreased, which was consistent with the results of the literature [40]. It can be seen that the abundance of *Firmicutes* and *Campylobacterota* decreased after JJSM treatment. After treatment with SASP and JJSM, the abundance of *Bacteroidetes* was significantly increased, especially in the low-concentration JJSM intervention groups. The above-mentioned changes made the intestinal flora structure of mice in the intervention group closer to the flora of the Control group. Accordingly, our study demonstrated that JJSM is beneficial to the recovery of beneficial bacteria and the inhibition of harmful bacteria to resist pathogen invasion to a certain extent.

**Figure 4:**
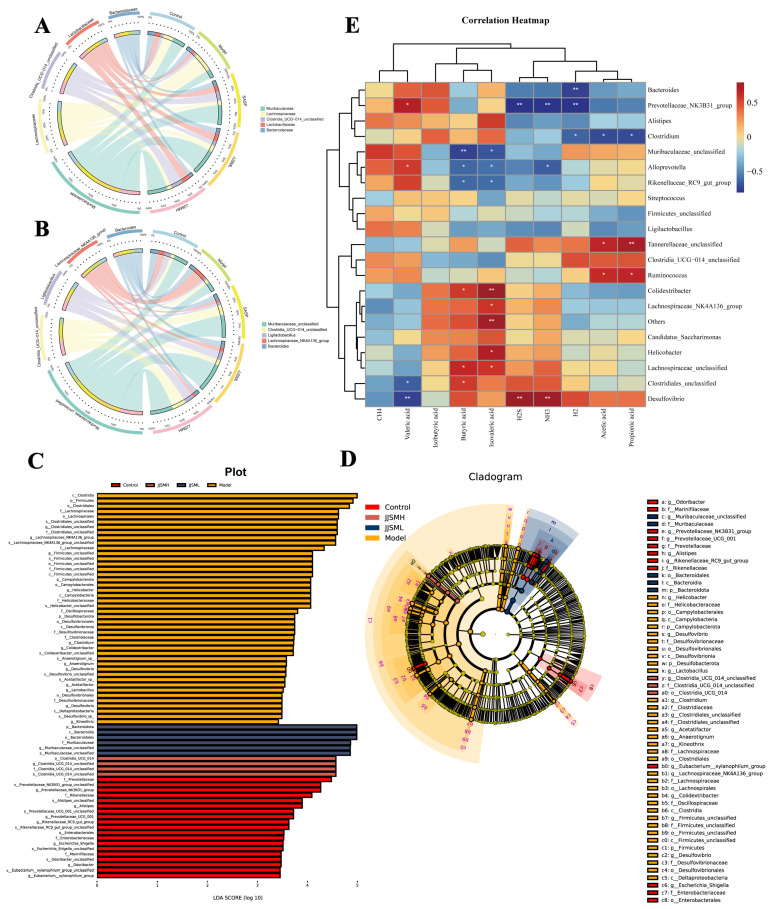
Circos diagram of species-sample relationships (top 5 bacteria). (**A**) At family level; (**B**) At genus level. LDA Effect Size (LEfSe): (**C**) Histogram of linear discriminant analysis (LDA) value distribution. LDA score ≥ 3.5. The length of the bar chart represents the size of the impact of significantly different species. (**D**) Cladogram. Each node’s size reflects the species’ relative abundance. (phylum = p; class = c; order = o; family = f; genus = g; species = s). (**E**) Microbiota and index correlation clustering heat map. Rows indicate species, and columns indicate gas and SCFAs relevant to this study. * *p* < 0.05, ** *p* < 0.01. Red indicates a positive correlation, blue indicates a negative correlation. The darker the color, the stronger the correlation.

To further reveal the specific genera that metabolize JJSM, the genus level in the samples was analyzed (Figure 3G). The relative abundance of beneficial bacteria *Muribaculaceae_unclassified* (Figure 3H), *Ligolactobacillus* (Figure 3I) and *Ruminococcus* (Figure 3J) decreased. *Muribaculaceae_unclassified* and *Ligolactobacillus* were among the most abundant (the top five) bacteria at the genus level, which played an important role in the overall coordination effect of the intestinal flora of mice. The physiological structure of the mucosal surface and immune cells constitute the mucosal barrier, the first line of defense of the intestinal immune system. When the intestinal mucosa is stimulated to a certain extent, intestinal mucus is produced to protect the intestine. At the family level, based on the existing literature, *Muribaculaceae* has been associated with colonic mucus thickness [41], and the abundance is positively correlated with the immune barrier [42]. In addition, the isomeride of *Muribaculaceae* has been shown to produce SCFAs, and their abundance is positively correlated with body weight, which may contribute to the maintenance of colon function and structural integrity [43]. After modeling, the abundance of this bacteria decreased, the intestinal wall was thickened and damaged in many places, and mice exhibited weight loss. The total SCFAs in this group were significantly lower than those in the control group. Overall, the results of this experiment were consistent with the previous literature [43]. The abundance of the bacteria was significantly improved in the JJSM intervention groups, indicating that JJSM repaired the immune barrier of colitis mice to a certain extent and accelerated inflammation recovery. At the genus level (Figure 4B), *Ligilactobacillus* in model mice was reduced. *Ligilactobacillus* helped resist adverse gastrointestinal diseases, improve intestinal barrier function, and reduce intestinal histological changes [44]. Moreover, the colonic tissue structure of the colitis mice was destroyed, with significant inflammatory cell infiltration, and hemorrhage occurred, indicating that the inflammatory response could not be alleviated. The JJSM intervention altered the proportion of the bacteria in the intestinal flora species, with pathological improvements observed at the tissue level, and inflammation was relieved to a large extent. Our findings demonstrated that JJSM could regulate the composition of the microbiota and have a restoring effect on the intestinal microbiota of DSS-induced colitis mice. Current evidence suggests that the combination of Albuca Bracteate Polysaccharide (ABP) and 5-Fluorouracil (5-FU) in CRC can enrich the gut microbiota, especially for *Ruminococcus,* and increase acetic acid, while propionic acid and butyric acid help to enhance the therapeutic effect of CRC [12]. In addition, an increasing body of evidence suggests that *Ruminococcus* is involved in the carbohydrate fermentation of human SCFAs, and the reduction of SCFAs is related to the reduction of the bacterial population [45], consistent with our results. Moreover, acetic acid and propionic acid showed a significant positive correlation.

The relative abundance of harmful bacteria *Clostridiales_unclassified* (Figure 3K), *Lachnospiraceae_NK4A136_group* (Figure 3L) and *Desulfovibrio* (Figure 3M) in colitis mice greatly increased; likewise, their abundances were further restored after JJSM intervention. *Lachnospirillum* has been shown to affect host health by producing acetate and butyrate, facilitating colonization resistance to enteric pathogens [7]. Significant negative correlation has been shown between *Lachnospiraceae_NK4A136_group* and intestinal permeability and plasma LPS levels in diet-induced-obese mice, and this group also exhibited a significant negative correlation with colon length [46]. In this experiment, the abundance of *Lachnospiraceae*, the overall length of the colon, and the results of acetic acid and butyric acid in colitis mice were consistent with the literature, and the relative abundance of the bacteria was greatly reduced after JJSM intervention.

It is worth mentioning that the abundance of *Desulfovibrio* in colitis mice was increased compared to the controls (Figure 3M), and *Desulfovibrio* is implicated in the production of intestinal gas H_2_S [22]. The final product of sulfate-reducing bacteria (SRB) in the gut is hydrogen sulfide, and an increase in the abundance of SRBs in the intestinal tract is thought to be a strong risk factor for IBD in animals and humans; this facilitates the development of colitis, which is closely related to the production of H_2_S [47]. The relative abundance of *Desulfovibrio* and H_2_S generation increased in our experiment, consistent with the reports indicating that this harmful bacterium is one of the direct causes of colitis in mice, and JJSM intervention could effectively reverse this.

#### 3.6.3. Overall Structural Regulation of Intestinal Microbiota after JJSM Treatment

The top five bacteria at the family and genus levels from the overall structural composition were used to understand the differences between the main bacterial groups. At the family level, an inter-group analysis with *Muribaculaceae*, *Lachnospiraceae*, *Lactobacillaceae*, *Clostridia_UCG-014_unclassified* and *Bacteroidaceae* in each group was performed (Figure 4A). The relative abundance of *Lachnospira* decreased from 13.02% in normal mice to 8.92% in colitis mice, consistent with the literature [48]. At the same time, the relative abundance of *Lactobacillaceae* was also reduced from 14.57% in normal mice to 4.88% in colitis mice, substantiating that *Lactobacillaceae* was associated with the anti-inflammatory effects [49]. In addition, while the abundance of *Muribaculaceae* was decreased in the model mice, the abundance of *Clostridia* was increased, and the abundance of these two bacteria was improved to varying degrees in the JJSM intervention groups. This indicated that JJSM repaired the immune barrier of colitis mice to a certain extent. At the genus level (Figure 4B), the genus *Lachnospiraceae_NK4A136_group* (thick-walled phylum) was enriched in UC mice, while *Muribaculaceae_unclassified* and *ligilactobacillus* were significantly decreased.

LDA Effect Size (LEfSe) analysis was used to investigate discrepancies in intestinal microbiota composition from phylum to genus for all groups of mice (Figure 4C,D). The dominant bacterial taxa changed significantly after DSS intervention. At the family level, the relative abundances of *f_Helicobacteraceae*, *f_Oscillospiraceae, f_Desulfovibrionaceae* and others were significantly increased in colitis mice. In addition, colitis mice had significantly higher abundances of *g_Helicobacter*, *g_Clostridium*, *g_Desulfovibrio* and other genera in colitis mice. However, JJSM and SASP groups exhibited significantly reduced abundances of important bacterial families and genera. A high concentration of *Oscillospiracea* in the intestinal flora of depression patients has been found [50], and thus the inflammation theory is important in the pathogenesis of depression. Changes in the gut microbiota suggested a significant correlation between the functions of the digestive, nervous and immune systems [26]. *Helicobacteraceae* has a well-recognized effect on the microbial structure of the gastric community, and several studies demonstrated that it could alter the distal gut microbiota [51]. The most important pathway may be intragastric hypochlorite caused by *Helicobacteraceae*, while sodium hypochlorite may facilitate the entry of acid-sensitive bacteria into the distal gastrointestinal tract, leading to changes in colonic microbiota [7]. *Helicobacteraceae* rapidly colonizes the gastric epithelial surface via flagella-mediated motility and chemotaxis, resulting in various serious diseases. These enterobacteria were consistently found in colitis mice in our study.

### 3.7. Correlation Analysis

The relationship between the top 20 genera and indicators of in vitro fermentation was assessed using correlation analysis to help us better understand the specificity of JJSM metabolic genera. As shown in Figure 4E, the four gases (H_2_S, NH_3_, CH_4_, H_2_) with high relative content in gas production and significant indigenous differences among groups were positively regulated by *Desulfovibrio* and negatively regulated by *Prevotellaceae_NK3B31_group*, *Bacteroides*, *Clostridium* and *Alloprevotella*. Among them, *Desulfovibrio* was positively correlated with H_2_S and NH_3_ and negatively with valeric acid. We also found that *Ruminococcus* and *Tannerellaceae_unclassified* were positively correlated with acetate propionate, whereas *Clostridium* was negatively correlated. The increase in *Clostridium* concentration in colitis mice was accompanied by an increase in butyrate concentration, and the increase in *Desulfovibrio* was accompanied by an increase in H_2_S content, which was consistent with our above results and further demonstrated our hypothesis. In addition, *Clostridium* and butyrate were positively correlated. Furthermore, in our study, the concentration of butyrate (*p* < 0.01) and the abundance of *Clostridium* (*p* < 0.01) were significantly increased in colitis mice, while the high concentration of butyrate [39] in colitis mice decreased the ability of pathogen clearance to a certain extent.

### 3.8. Microbiota Functional Prediction

To compare the functional potential of bacteria, we predicted functional signatures in the UC microbiota by using the Phylogenetic Investigation of Communities by Reconstruction of Unobserved States (PICRUSt2) as a predictive exploration tool. We selected level 3 biological pathways from the Kyoto Encyclopedia of Genes and Genomes (KEGG) to predict metabolic functions based on microbiota metagenomics [52]. According to metabolomics studies, numerous metabolic pathways may be linked to the number of bacteria [53].

**Figure 5:**
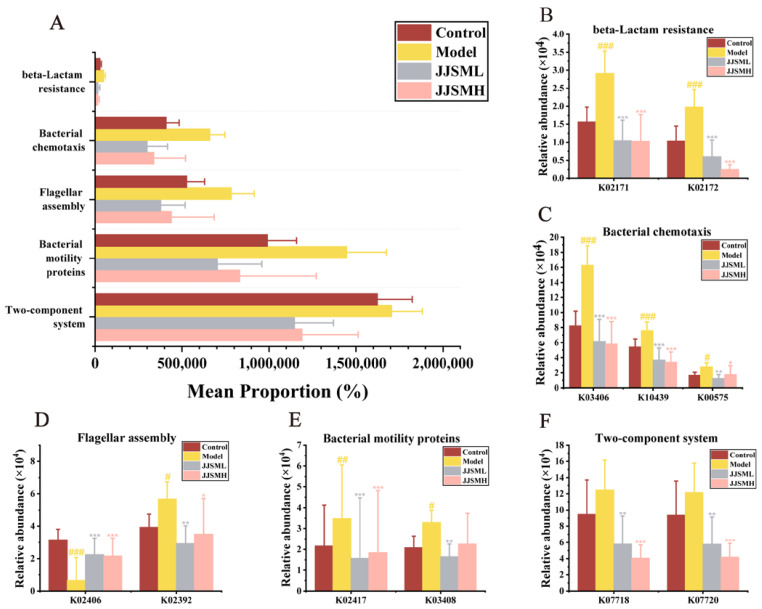
Function prediction. (**A**) Level 3 KEGG pathway alterations. KOentries with dominant abundance and significant differences in the pathway. (**B**) Beta-Lactam resistance; (**C**) Bacterial chemotaxis; (**D**) Flagellar assembly; (**E**) Bacterial motility proteins; (**F**) Two-component system. ^#^ *p* < 0.05 vs. control, ^##^ *p* < 0.01 vs. control, ^###^ *p* < 0.001 vs. control, * *p* < 0.05 vs. model, ** *p* < 0.01 vs. model, *** *p* < 0.001 vs. model.

The KEGG pathway analysis (Figure 5A and Table S2) showed significant enrichment in the Two-component system, Bacterial motility proteins, Flagellar assembly, Bacterial chemotaxis and beta-Lactam resistance in colitis mice. We screened the relevant KOentries according to their abundance in the pathways that may be involved in inflammation and then carried out secondary screening according to the significance of differences from the KOentries with the most abundant abundance to obtain the relevant changes of the corresponding proteins or factors of each pathway.

Motility is a crucial factor for the survival of bacteria in the gastrointestinal tract representing adaptive responses to various stimuli. Motile bacteria mainly use the flagella to efficiently direct their motility or chemotaxis to transfer to a more favorable environment. Our results suggested that the abundance of *Campylobacter* was significantly increased in colitis mice (Figure 3F) and that bacterial motility was most likely enhanced in DSS-induced colitis by enhancing the flagellar assembly pathway. *Campylobacter* has been shown to exhibit unusual motility, especially in sticky substances [54], which also confirmed that the pathogenesis of colitis involved bacterial chemotaxis and the participation of bacteria containing bacterial motor proteins. The strong motility of flagellate bacteria leads to the colonization of many opportunistic pathogens in the epithelium, resulting in intestinal environment disorder. Many symbiotic microbiotas exist in the outer mucosa of the large intestinal epithelial cells [55], and the intestinal wall can be invaded by commensal and pathogenic microorganisms, resulting in a compromised barrier. It was found that the BlaI family transcription factors, penicillinase inhibitor and regulatory protein blaR1, were upregulated in beta-lactam resistance in colitis (Figure 5B). The methyl-accepting chemotaxis protein and ribose transport system substrate-binding protein were upregulated and the chemotactic protein methyltransferase CheR was downregulated (Figure 5C) in bacterial chemotaxis. Moreover, flagellin and flagellar basal-body rod protein FlgG were upregulated (Figure 5D) in flagellar assembly and flagellar motor switch protein FliN/FliY, and purine-binding chemotaxis protein CheW (Figure 5E) were upregulated in bacterial motility proteins. Finally, the sensor histidine kinase YesM and the response regulator YesN were upregulated in the two-component system (Figure 5F). Significant differences were observed between the JJSM drug intervention groups and the model group, indicating that JJSM exerted its inhibitory effect on colitis mainly through the upregulation and downregulation of these cytokines and proteins.

### 3.9. Mechanism and Prospect of JJSM Improving Intestinal Microecology in Colitis Mice

**Figure 6:**
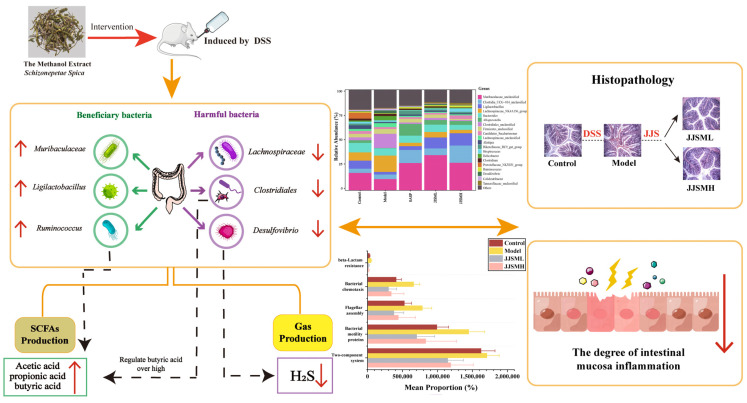
Schematic diagram of JJSM relieving colitis.

The outcomes of this research suggested that gas-mediated intestinal microbiome regulation prompted the JJSM to relieve DSS-induced colitis (Figure 6).

JJSM reversed the intestinal dysbiosis and its regulatory effect upregulated the abundance of beneficial bacteria such as *Muribaculaceae_unclassified*, *Ligolactobacillus*, *Ruminococcus*, etc., promoting the generation of SCFAs and strengthening the intestinal immune barrier. In this respect, *Ruminococcus*-specific enrichment increased the beneficial SCFAs, acetic, propionic and butyric acids, enhancing the therapeutic effect against colitis and even colorectal cancer.

In addition, JJSM downregulated harmful bacteria such as *Clostridiales*, *Lachnospiraceae_NK4A136_group* and *Desulfovibrio*, which reduced the colonization ability of harmful bacteria in the intestine and the secondary damage caused by them, and improved the disturbance of intestinal homeostasis, thereby alleviating the symptoms of colitis. Importantly, the anaerobic bacteria *Desulfovibrio* could reduce sulfate to produce H_2_S. H_2_S exhibits cytotoxic and proinflammatory properties, which can disrupt the stability of the protective mucous layer and induce epithelial cell damage and local intestinal inflammation. Likewise, JJSM intervention significantly reduced the abundance of *Clostridiales*, regulated the high concentration of butyric acid and repaired the intestinal barrier, alleviating intestinal inflammation. JJSM mainly inhibited the production of H_2_S and reduced excessive acetic acid concentration by reducing the abundance of *Desulfovibrio* and *Clostridiales*, thereby improving intestinal wall damage, attenuating superficial colonic ulcers and relieving colitis symptoms.

According to the results and PICRUSt2, the two-component system, Bacterial Motility proteins, Flagellar Assembly, Bacterial Chemotaxis, and beta-lactam Resistance to several possible pathways for the formation of ulcerative colitis in mice were predicted. The adjustment of microbiota structure restored the functions of cytokines and proteins related to these metabolic pathways. However, the therapeutic potential and mechanism of action of the key strains and individual active compounds of JJSM, which exert anti-inflammatory activity and its individually dependent metabolites, warrant further exploration. Our future studies will focus on the effectiveness and mechanism of medication intervention in the development of CRC cancer from UC.

## 4. Conclusions

This study provides evidence on the impact of JJSM in DSS-induced colitis mice and demonstrates how JJSM alleviates DSS-induced colitis by regulating intestinal gas and SCFAs and restoring the imbalance of intestinal flora structure; JJSM (including anti-inflammatory compounds such as luteolin, eriodictyol, fisetin and kaempferol) relieves inflammation by ameliorating the disease phenotype of colitis mice, severity of damage and lesion range of the colon, which can prevent the overgrowth of harmful bacteria like *Lachnospiraceae*, *Clostridiales* and *Desulfovibrio* caused by the imbalance of intestinal flora after DSS induction and reduce the risk of massive H_2_S production. The upregulation of beneficial bacteria such as *Ligilactobacillus*, *Muribaculaceae*, etc., improves the intestinal microenvironment and relieves the inflammatory response caused by the destruction of the intestinal barrier. Regarding the effect of JJSM on improving intestinal microecology and intestinal barrier, we speculate that *Schizonepetae Spica* has huge prospects as an anti-ulcerative colitis drug. However, the therapeutic potential and mechanism of individual active compounds of *Schizonepetae Spica*, which exert anti-inflammatory activity, to target the key strains metabolites in mice with ulcerative colitis intervention, warrant more exploration. Further studies are scheduled to investigate these questions. Indeed, our future studies will focus on the specific mechanism of JJSM on UC and explore its potential activity against CRC development.

## Figures and Tables

**Table 1:** Characterization of chemical constituents in JJSM.

No.	RT/min	*m*/*z*	Type	Formula	Identify	OB/%	DL	Class
1	9.39	283.0618	[M − H]^−^	C_16_H_12_O_5_	Glycitein	50.48	0.24	Isoflavonoids
2	9.82	285.0397	[M − H]^−^	C_15_H_10_O_6_	Luteolin	36.16	0.25	Flavonoids
3	9.50	285.0397	[M − H_2_O − H]^−^	C_15_H_12_O_7_	Taxifolin	57.84	0.27	Flavonoids
4	11.56	285.0435	[M − H]^−^	C_15_H_10_O_6_	Kaempferol	41.88	0.24	Flavonoids
5	10.43	287.0542	[M + H]^+^	C_15_H_10_O_6_	Fisetin	52.6	0.24	Flavonoids
6	10.40	289.0705	[M + H]^+^	C_15_H_12_O_6_	Eriodictyol	71.79	0.24	Flavonoids
7	8.52	301.073	[M − H]^−^	C_16_H_14_O_6_	Hesperetin	70.31	0.27	Flavonoids
8	11.46	299.0564	[M − H]^−^	C_16_H_12_O_6_	Kaempferide	73.41	0.27	Flavonoids
9	10.22	308.2191	[M + H]^+^	C_18_H_29_NO_3_	Dihydrocapsaicin	47.07	0.19	Benzene derivatives

## Data Availability

All consensus sequence data of mice were submitted to the National Center for Bio-technology Information Short Read Archive under accession no. PRJNA865047.

## Supplementary Materials

The following supporting information can be downloaded at: https://www.mdpi.com/article/10.3390/nu15030519/s1. Table S1: Effects of JJSM on overall structural modulation of gut microbiota; Table S2: Correlation between KO entry and KEGG Level 3 related to colitis; Table S3: Abbreviations.
