# Supplementary material for: Gas-Mediated Intestinal Microbiome Regulation Prompts the Methanol Extract of Schizonepetae Spica to Relieve Colitis
Source: Nutrients. 2023 Jan 19;15(3):519. doi: 10.3390/nu15030519 (PMC9921049; doi:10.3390/nu15030519)
Supplement: Supplementary file 1 [file nutrients-15-00519-s001.zip › nutrients-2079964-supplementary.pdf]

Table S1: Effects of JJSM on overall structural modulation of gut microbiota

	Control	Model	SASP	JJSM-L	JJSM-H
Goods_coverage	1	1	1	1	1
Chao1	611.065±177.315	509.21±219.04	358.915±206.915	608.59±199.98	452.83±189.95
Shannon	6.77±0.35	6.435±0.475	5.6±0.570	6.355±0.915	5.995±0.775

Table S2: Correlation between KO entry and KEGG Level 3 related to colitis

function ID	description	Control	Model	SASP	JJSM-L	JJSM-H
K00575	cheR; chemotaxis protein methyltransferase CheR	16652.31	28061.10	14969.81	12487.04	17603.96
K02171	blaI; BlaI family transcriptional regulator, penicillinase repressor	15644.74	29145.57	11852.95	10492.1	10317.59
K02172	blaR1; bla regulator protein blaR1	10393.59	19784.54	5418.38	6014.386	2506.24
K02392	flgG; flagellar basal-body rod protein FlgG	39313.06	56791.85	31657.21	29315.11	35116.19
K02406	fliC; flagellin	31590.74	65474.34	22093.50	22495.36	21667.83
K02417	fliNY, fliN; flagellar motor switch protein FliN/FliY	21639.70	34739.27	16599.67	15736.13	18467.50
K03406	mcp; methyl-accepting chemotaxis protein	82279.18	162689.42	59616.19	61718.53	58103.60
K03408	cheW; purine-binding chemotaxis protein CheW	20823.16	32907.65	18836.77	16537.97	22606.35
K07718	yesM; two-component system, sensor histidine kinase YesM	94571.38	124679.02	55823.14	58111.4	40758.10
K07720	yesN; two-component system, response regulator YesN	93599.84	121584.18	55869.17	57857.15	41928.32
K10439	rbsB; ribose transport system substrate-binding protein	54150.38	75967.16	35101.51	36925.53	34032.60

Table S3: Abbreviations

Abbreviations	Full name
UC	ulcerative colitis
JJSM	*Schizonepetae Spica*
AhR	aryl hydrocarbon receptor
Nrf2	nuclear factor-erythroid 2 related factor 2
TLR4	Toll-like receptor 4
DAI	disease activity index
SCFAs	short-chain fatty acids
DSS	dextran sulfate sodium
LC-MS	liquid chromatography-mass spectrometry
SPF	specific pathogen-free
SASP	Salazosulfapyridine
H&E	Hematoxylin and Eosin
CO_2	carbon dioxide
H_2	hydrogen
CH_4	methane
H_2S	hydrogen sulfide
NH3	ammonia
DADA2	Divisive Amplicon Denoising Algorithm
LDA	linear discriminant analysis
LEfSe	LDA Effect Size
TCMSP	traditional Chinese medicine systems pharmacology
OB	oral bioavailability
DL	drug-like properties
NMDS	non-metric multidimensional scaling
PICRUSt2	Phylogenetic Investigation of Communities by Reconstruction of Unobserved States
KEGG	Kyoto Encyclopedia of Genes and Genomes
IBD	inflammatory bowel disease
DIO	diet-induced-obese
SRB	sulfate-reducing bacteria
ABP	Albuca Bracteate Polysaccharide
5-FU	5-Fluorouracil.
CRC	Colon and Rectal Cancer
